# 放疗联合免疫治疗非小细胞肺癌：前沿学术问题专家交流共识

**DOI:** 10.3779/j.issn.1009-3419.2020.102.24

**Published:** 2020-06-20

**Authors:** 放射肿瘤学免疫联合治疗专家讨论小组, 星浩 艾, 勇 蔡, 倩 褚, 琤波 韩, 铀 卢, 颂兵 秦, 麟 邬, 丛华 谢, 智勇 袁, 文昭 钟, 晓霞 朱, 玉蛟 张, 正飞 朱

**Affiliations:** 1 200030 上海，上海交通大学医学院上海市胸科医院肺部肿瘤临床医学中心 Lung Tumor Clinical Medical Center, Shanghai Chest Hospital, Shanghai Jiao Tong University, Shanghai 200030, China; 2 200433 上海，同济大学附属上海市肺科医院放疗科 Department of Radiation Oncology, Shanghai Pulmonary Hospital, Tongji University School of Medicine, Shanghai 200433, China; 3 430030 武汉，华中科技大学同济医学院附属同济医院 Department of Radiation Oncology, Tongji Hospital, Tongji Medical College, Wuhan 430030, China; 4 110022 沈阳，中国医科大学附属盛京医院肿瘤科 Department of Clinical Oncology, Shengjing Hospital, China Medical University, Shenyang 110022, China; 5 610041 成都，四川大学华西医院肿瘤科 Department of Oncology, West China Hospital, Sichuan University, Chengdu 610041, China; 6 215006 苏州，苏州大学附属第一医院放疗科 Department of Radiation Oncology, The First Affiliated Hospital of Suzhou University, Suzhou 215006, China; 7 410013 长沙，中南大学湘雅医学院附属湖南省肿瘤医院 Hunan Cancer Hospital The Affiliated Cancer Hospital of Xiangya School of Medicine, Central South University, Changsha 410013, China; 8 430071 武汉，武汉大学中南医院肿瘤放化疗科 Department of Cancer Radiotherapy and Chemotherapy, Zhongnan Hospital of Wuhan University, Wuhan 430071, China; 9 300060 天津，天津医科大学肿瘤医院放疗科 Department of Radiation Oncology, Cancer Institute and Hospital, Tianjin Medical University, Tianjin 300060, China; 10 510080 广州，广东省肺癌研究所 广东省人民医院 广东省医学科学院 Cancer Center, Guangdong General Hospital, Guangdong Academy of Medical Sciences, Guangdong Lung Cancer Institute, Guangzhou 510080, China; 11 510515 广州，南方医科大学南方医院放疗科 Department of Radiation Oncology, Nanfang Hospital, Southern Medical University, Guangzhou 510515, China; 12 TX 77030 休斯顿，德克萨斯大学MD Anderson肿瘤中心放疗科 Department of Radiation Oncology, The University of Texas MD Anderson Cancer Center, Houston TX 77030, USA; 13 200032 上海，复旦大学附属肿瘤医院放疗科 Department of Radiotherapy, Fudan University Shanghai Cancer Center, Shanghai 200032, China

**Keywords:** 免疫检查点抑制剂, 肿瘤, 放疗, 免疫治疗, 不良反应, 毒性, 混合反应, 脑转移, Immune checkpoint inhibitors, Neoplasms, Radiotherapy, Immunotherapy, Adverse effects, Toxicity, Mixed responses, Brain metastases

## Abstract

肺癌是目前导致全球和中国癌症患者死亡的主要瘤种。多年来，常规的肿瘤治疗方法，如手术、化疗和放疗一直主导着非小细胞肺癌（non-small cell lung cancer, NSCLC）治疗领域。临床实践中引入免疫疗法使肺癌的治疗与其他实体瘤一样发生了根本性转变。最新临床前和临床数据表明，放疗可以通过诱导免疫原性细胞死亡和重新编程肿瘤微环境促进抗肿瘤免疫反应。研究者开始重新审视放疗作为免疫治疗的联合疗法，导致研究其潜在协同作用的临床试验数量呈指数级增长。放疗联合免疫治疗的临床试验引起了医疗界的广泛关注，会议邀请专家交流前沿及争议学术问题：①放疗联合免疫检查点抑制剂治疗NSCLC最新进展；②放疗联合免疫治疗是否显著增加毒性；③免疫检查点抑制剂治疗后出现的混合反应及局部治疗的干预价值；④放疗联合免疫治疗脑转移瘤的机制和进展。

## Abstract (en)

Lung cancer is the leading cause of cancer death worldwide as well as in China. For many years, conventional oncologic treatments such as surgery, chemotherapy, and radiotherapy (RT) have dominated the field of non-small cell lung cancer (NSCLC). The recent introduction of immunotherapy in clinical practice, led to a paradigm shift in lung cancer as in many other solid tumors. Recent pre-clinical and clinical data have shown RT may also modify antitumor immune responses through induction of immunogenic cell death and reprogramming of the tumor microenvironment. This has led many to reexamine RT as a partner therapy to immuno-oncology treatments and investigate their potential synergy in an exponentially growing number of clinical trials. Clinical trials combining radiotherapy and immunotherapy are attracting major attention, experts were invited to discuss frontier and controversial academic topics: (1) Recent developments of clinical synergy between radiation and immune checkpoint inhibitors (ICIs) in the treatment of NSCLC; (2) Will immunotherapy and radiotherapy increase the toxicity risk for cancer patients; (3) How to cope the mixed responses/disassociated responses phenomenon in checkpoint inhibition therapy to NSCLC with local ablative therapy; (4) Combining radiotherapy and immunotherapy in the treatment of NSCLC brain metastases.

免疫治疗是继手术、放化疗、靶向药物治疗后又一有效肿瘤治疗手段。免疫检查点抑制剂（immune checkpoint inhibitors, ICIs）是其中最成功的治疗方法之一，因其疗效确切，应答长期持久，已获批应用于黑色素瘤、霍奇金淋巴瘤、膀胱癌等多个瘤种。晚期NSCLC治疗领域，程序性死亡受体-1（programmed cell death-1, PD-1）和程序性死亡受体-配体-1（programmed cell death-ligand 1, PD-L1）抑制剂已获美国食品药品管理局（Food and Drug Administration, FDA）批准进入临床应用，国内多个ICIs也先后获得国家药品监督管理局批准进入临床。随着PACIFIC研究^[1-3]^和PEMBRO-RT研究^[4]^公布，放疗联合免疫治疗已成为当前的热门研究领域之一^[5]^。尽管联合方案的协同增效作用通过大型Ⅲ期临床研究得到证实，临床医生在实际应用中仍存在很多疑问，特别是对于一些存在争议的问题，亟需更多有效信息。放射肿瘤学免疫联合治疗专家讨论组邀请来自MD Anderson肿瘤中心放疗专家张玉蛟教授以及来自国内多家肿瘤治疗中心的专家教授以网络连线专家讨论的形式就以下问题展开深入交流探讨：①放疗联合免疫治疗非小细胞肺癌（non-small cell lung cancer, NSCLC）最新进展；②放疗联合免疫治疗是否显著增加毒性；③ICIs治疗后出现的混合反应及局部治疗的干预价值；④放疗联合免疫治疗脑转移瘤的机制和进展讨论。专家讨论组试图总结出对指导临床实践有帮助的意见共识。

## 1 放疗联合免疫治疗研究进展

相较免疫治疗，放疗联合免疫治疗在多项临床试验中显著提升了患者治疗的有效率，颠覆放疗杀伤免疫细胞，具有免疫抑制作用的传统观点，提示放疗和免疫治疗的协同作用^[6]^。放疗可以通过多种方式影响肿瘤的免疫状态，如促进肿瘤细胞释放肿瘤特异性抗原，提高免疫系统的杀伤能力^[7]^，而免疫治疗的加入，可以进一步促进这一系列过程甚至增加“远隔效应”的发生率^[8, 9]^。

### 1.1 放疗联合免疫治疗作用机制

放疗可能通过直接杀伤和诱导肿瘤细胞免疫源性死亡，调节肿瘤细胞表型，并且使肿瘤血管正常化以及促进免疫细胞浸润和系统治疗药物局部浸润，来达到与免疫治疗协同增效的作用^[6-8]^：①放疗可以促进高迁移率族蛋白B1（high mobility group box 1, HMGB1）、二磷酸腺苷（adenosine diphosphate, ADP）和尿酸等释放，并且通过刺激钙网蛋白运输到细胞表面，促进肿瘤细胞免疫源性死亡（immunogenic cell death, ICD）；②放疗导致蛋白质分解增加，诱导肿瘤细胞表面主要组织相容性复合体Ⅰ类（major histocompatibility complex I, MHC I）蛋白质的负载和表达增加，促进细胞毒性T细胞识别肿瘤相关抗原（tumor-associated antigens, TAAs）；③放疗促进细胞质DNA积累，通过干扰素基因刺激蛋白（interferon genes protein, STING）和环状GMP-AMP合成酶（cyclic GMP-AMP synthase, cGAS）途径的激动剂诱导免疫激活；④肿瘤细胞死亡后释放细胞碎片中的损伤关联分子模式（damage-associated molecular patterns, DAMPs）、TAAs和炎性细胞因子，激活抗原呈递细胞，如树突状细胞，向淋巴结中的免疫细胞呈递TAAs。多克隆TAAs特异性细胞毒性T细胞被激活，杀伤放疗照射局部和远位肿瘤，免疫治疗可增强这种反应，并且奠定了放疗与免疫治疗联合应用的基础。局部放疗可激活免疫系统，诱导免疫细胞对放疗区域以外的肿瘤细胞进行攻击，这种现象被称为“远隔效应”。临床单独放疗产生远隔效应少见，免疫治疗可增强放疗免疫诱导效应，增加远隔效应的发生率^[10, 11]^。放疗与免疫治疗协同抑制肿瘤生长，达到1+1 > 2的效果^[12]^。

### 1.2 放疗联合免疫治疗在NSCLC患者中显示出一定的疗效改善

2017年*Lancet Oncology*杂志发表了一项针对KEYNOTE-001研究中接受帕博利珠单抗单药治疗的晚期NSCLC患者的单中心回顾性分析^[13]^，评价了帕博利珠单抗治疗前是否接受放疗对意向治疗人群的无进展生存时间（progression-free survival, PFS）和总生存时间（overall survival, OS）的影响。结果显示，帕博利珠单抗治疗之前接受放疗的患者，相比未接受放疗的患者，总生存显著延长（中位OS: 10.7个月*vs* 5.3个月，HR=0.58，*P*=0.026；中位PFS: 4.4个月*vs* 2.1个月，HR=0.56，*P*=0.019）。这一分析结果初步提示放疗可改善晚期肿瘤患者后线免疫治疗效果。

在前瞻性PEMBRO-RT Ⅱ期研究中^[4]^，立体定向放疗（stereotactic body radiation therapy, SABR）序贯帕博利珠单抗组相比帕博利珠单抗单药组12周时的总缓解率（overall response rate, ORR）提高1倍（36% *vs* 18%, *P*=0.07），患者中位总生存时间由单用免疫治疗的7.6个月延长至15.9个月（HR=0.66, *P*=0.16）。尽管ORR未达预先制定的临床终点标准，在晚期转移性NSCLC治疗中，二线局部放疗联合免疫治疗的协同作用结果仍值得肯定。

放疗联合免疫在晚期NSCLC患者中取得了积极结果，对于不可手术切除的局部晚期NSCLC患者带来临床改善的可能。PACIFIC研究^[3]^是一项随机、双盲、安慰剂对照的国际多中心Ⅲ期临床研究，旨在评估度伐利尤单抗在用于经含铂方案同步放化疗后，未发生疾病进展的不可手术的Ⅲ期NSCLC患者巩固治疗的疗效。研究显示同步放化疗联合免疫的研究组OS具有显著优势（HR=0.69, 95%CI: 0.55-0.86），研究组中位OS仍未达到（95%CI: 38.4-NR），而对照组中位OS为29.1个月（95%CI: 22.1-35.1）。研究组1年、2年和3年总生存率均高于对照组（83.1% *vs* 74.6%，66.3% *vs* 55.3%和57.0% *vs* 43.5%）。基于PACIFIC研究，同步放化疗后序贯巩固度伐利尤单抗已被美国国立综合癌症网络、欧洲肿瘤内科学会、中国临床肿瘤学会列为Ⅲ期NSCLC的标准治疗方案。同样采用同步放化疗后序贯免疫治疗的Ⅱ期单臂LUN14-179研究^[14]^显示，对于不可切除Ⅲ期NSCLC患者同步放化疗之后帕博利珠单抗巩固治疗，患者的中位至疾病转移或死亡时间（time to metastatic disease or death, TMDD）提高至22.4个月，中位PFS为17.0个月。包括纳武利尤单抗（CTR20200425）、度伐利尤单抗（CTR20181576）、信迪利单抗（NCT03884192）、CS-001（CTR20181429）在内的多个ICIs正在国内进行Ⅲ期NSCLC同步放化疗联合的随机对照研究。

另一项针对局部晚期NSCLC的Ⅱ期临床研究DETERRED^[15]^中，同步组将免疫治疗时间进一步前移，同步放化疗阶段给予阿特珠单抗治疗，初步有效性分析显示中位PFS为13.2个月，中位OS尚未达到，而单纯同步放化疗后巩固化疗联合阿特珠单抗的序贯组中位PFS为18.6个月，中位OS为22.8个月。同步方案相比序贯方案能否取得生存获益，仍需通过更成熟、更大规模的临床研究验证。

## 2 放疗联合免疫治疗的安全性

基于上述临床证据，放疗联合免疫治疗的前景值得期待。但联合治疗模式是否增加3级以上不良反应的发生率，将决定该模式在临床实践的可行性。随着ICIs进入肿瘤治疗临床实践，临床医生对治疗相关性不良反应/免疫相关性毒性逐渐积累了更多的经验和认识。但到目前为止，对于免疫相关不良反应（immune-related adverse effects, irAEs）发生的明确的病理机制仍然处在探索阶段，可能的机制包括免疫治疗能够提高T细胞对正常组织表达的自身抗原的应答^[16]^，增加自身免疫抗体的表达，增加免疫因子的分泌和释放，增加补体介导的免疫应答。作为靶标的免疫检查点，细胞毒性T淋巴细胞相关抗原-4（cytotoxic T lymphocyte associated antigen-4, CTLA-4）/PD-1在正常组织中表达并参与免疫稳态的形成，比如CTLA-4在脑垂体上有表达。放疗和ICIs协同调节免疫应答的机制也可能影响治疗相关不良反应的类型和严重程度。一系列回顾性和少数前瞻性单臂/随机化研究的数据，提供了大量的证据，表明姑息性放疗和ICIs的联合治疗总体安全，没有实质性的免疫特异性不良事件的增加。现有证据表明，在对Ⅲ期NSCLC进行根治性放化疗后，使用PD-L1/PD-1抑制剂不会增加3级肺炎的发病率。但有报道提示，高剂量立体定向颅内放射治疗脑转移瘤患者使用ICIs可能会增加治疗相关脑坏死的风险^[17]^。相较于免疫和放疗两者单独治疗，多项临床试验提示放疗联合免疫治疗未显著增加3级以上不良反应。放疗联合免疫治疗存在的不良反应会不会成为临床应用瓶颈，仍是目前被广泛关注的问题。

### 2.1 放疗联合免疫治疗未显著增加3级以上不良反应

一项回顾性研究^[18]^中，肺部立体定向放疗（stereotactic body radiation therapy, SBRT）同步ICIs治疗的所有级别不良反应发生率为33.9%，3级及以上不良反应发生率为10.7%，而单独SBRT治疗组所有级别不良反应发生率为27.9%，3级及以上不良反应发生率为0.0%，SBRT同步ICIs对比单独SBRT治疗安全可耐受（*P*=0.472），3级以上肺炎发生风险升高（*P*=0.007）。而另一项荟萃分析^[17]^显示放疗联合ICIs治疗未显著增加3级以上肺炎的发生风险，发生率大约在3%-4%，与安慰剂组相似。

PACIFIC研究^[1-3]^中度伐利尤单抗联合RT治疗，联合治疗组和单独RT对照组3级/4级任何原因不良事件发生率为29.9% *vs* 26.1%，所有级别肺炎发生率为33.9% *vs* 24.8%，主要表现为1级/2级肺炎，而3级/4级肺炎发生率为3.4% *vs* 2.6%。对比一项度伐利尤单抗研究（304例NSCLC队列）^[19]^中单纯免疫治疗导致的不良反应中3级/4级不良反应发生率为3%，任何级别肺炎发生率为2.6%，3级/4级肺炎发生率为0.3%。联合治疗的互作效应额外增加3级/4级不良反应发生率约0.8%，任何级别肺炎发生率约6.5%，3级/4级肺炎约0.5%，因此免疫联合放疗并没有显著增加3级/4级肺炎发生。

不同的放疗技术同样会影响不良反应的发生率。RTOG-0617研究^[20, 21]^证实调强放疗较普通三维适形放疗3级以上放射性肺炎发生率降低（7.9% *vs* 3.5%, *P*=0.039）。放疗模式对免疫联合治疗的安全性的影响仍然需要重点关注，治疗技术的进步、治疗水平的提高和相关经验的积累可降低不良反应的发生。

### 2.2 不同人群的肺部不良反应发生率存在差异

在不同族裔中，免疫治疗的不良反应可能存在差异。一项聚焦PACIFIC研究不同亚组肺部不良反应的分析^[22]^显示，亚裔人群肺炎发生率高于高加索人群（47.9% *vs* 17.6%），表皮生长因子受体（epidermal growth factor receptor, *EGFR*）突变阳性人群的肺炎发生率高于*EGFR*突变阴性人群（11.0% *vs* 3.8%），且以上结果与其治疗方案无关。尽管该研究中无论是在治疗组还是对照组，亚裔人群的间质性肺炎发生率均高于高加索人群，张玉蛟教授指出MD Anderson肿瘤中心临床治疗亚裔人群与高加索人群未见明显差异，考虑肺损伤可能还与环境因素有关，仍需进行更加严谨和针对性的研究，以明确这一差异，指导临床实践。

### 2.3 不同治疗时序的不良反应

DETERRED研究^[15, 23]^中，同步组相比序贯组的不良反应发生率没有增加，3级及以上不良反应发生率为80% *vs* 80%，3级及以上免疫相关不良反应发生率为20% *vs* 30%，2级及以上肺炎发生率为16% *vs* 10%。鉴于DETERRED是小样本非随机对照试验，进一步结论还需更多数据支持。同步治疗相对于序贯治疗的优势，目前仅在动物模型中得到验证，2014年在*Cancer Research*杂志发表了一项研究^[24]^评估CT26肿瘤小鼠接受不同的联合治疗方案的结局，相对单纯放疗组，同步治疗组总体生存率明显提高（未提供数据）；序贯治疗组与单纯放疗相比，未提高总体生存率（中位生存期为35 d *vs* 30 d，*P* > 0.05）。专家讨论组认为，如果患者没有哮喘、慢性阻塞性肺炎等相关基础疾病，可选择将免疫治疗提前与放化疗同步，不良反应可控，同时可能增加肿瘤治愈的机会。张玉蛟教授提出，在未来研究中可考虑在根治性放疗阶段去除化疗，使用放疗和免疫治疗同步或序贯治疗方案，提高疗效的同时减轻不良反应。

### 2.4 小结

综上，放疗联合免疫治疗不良反应发生率未显著增加，毒副反应处于可耐受范围。同步放化疗后序贯免疫治疗是目前不可手术的Ⅲ期NSCLC新的标准治疗模式，基于目前随机Ⅲ期对照临床研究的循证医学证据，同步放化疗联合免疫治疗能够明显延长患者生存。随着临床证据的增加，免疫治疗的时机将有望从Ⅲ期、Ⅳ期肺癌治疗逐渐推进到早期肺癌治疗。目前的研究方向一是提高放疗联合免疫治疗的疗效，减少不良反应，在早期同步放化疗阶段即联合免疫治疗的新模式。另一重要研究方向是早期同步方案使用ICIs替代化疗。

## 3 免疫治疗的混合反应（mixed response, MR）及局部治疗

MR在NSCLC的化疗和靶向治疗时代均有发生。38%携带*EGFR*突变的NSCLC可能存在对酪氨酸激酶抑制剂（tyrosine kinase inhibitors, TKIs）的高度异质性反应，即同时存在缓解病灶和进展病灶的情况，并提示该现象与不良预后相关。随着免疫治疗进入临床实践，在接受ICIs治疗的人群中也观察到同样的现象，免疫治疗时代MR的定义^[25, 26]^包括：①至少一个肿瘤病灶增大[以实体瘤的疗效评价标准（Response Evaluation Criteria in Solid Tumors, RECIST）标准判断进展]，而另一个肿瘤病灶缩小；②一个或多个肿瘤病灶保持稳定，而另一个肿瘤病灶增大；③一个或多个肿瘤病灶保持稳定，而另一个肿瘤病灶缩小；④出现新的肿瘤病灶，而其他肿瘤缩小或保持稳定。MR常常代表一种治疗困境，导致系统治疗中断或换药，尽管此时仍有病灶对原治疗方案敏感。

MR考虑与肿瘤的高度瘤内异质性有关。2019年发表于*Journal of Clinical Oncology*的一项报道^[27]^针对单个靶病灶接受免疫治疗NSCLC和错配修复缺陷（mismatch repair deficiency, MMRD）肿瘤应答的情况进行分析发现，单个转移病灶对治疗的应答时间-空间模式（开始应答时间、应答率）倾向一致，符合克隆特异性T细胞在外周动员迁徙和浸润在肿瘤病灶区域进行杀伤的假说。与此相反的是，混合进展中不同病灶（同一个患者）的缓解情况存在异质性，这种情况较为常见（NSCLC 45%, MMRD 53%）。2019年Mayo Clinic的Baldeo^[25]^报道了1例接受PD-1抑制剂治疗后出现MR的小细胞肺癌患者的有肿瘤应答病灶和无应答病灶基因组特征，应答病灶存在更高的肿瘤突变负荷（tumor mutation burden, TMB）及*SMAD2/4*扩增，提示肿瘤针对免疫治疗的基因异质性和耐药机制。随着接受免疫治疗患者增多，观察到的MR现象或将相应增加，针对MR的机制进一步研究也会得到更多信息。

### 3.1 免疫治疗MR的判断

根据肿瘤免疫治疗相关评价标准（modified RECIST 1.1 for immune based therapeutics, iRECIST）和免疫相关反应标准（immune-related response criteria, irRC）及免疫治疗规范，在免疫治疗时代并不能第一时间判断病灶情况为MR，倾向于等待下一次评估。评估时间的长短会影响对免疫治疗MR的判断。在免疫中位起效时间（4周-8周）之前去定义MR并不恰当^[28]^，应在接受第一次ICIs治疗后3个月左右做判断。同时需要注意免疫治疗过程中可能出现的假进展和超进展现象，对局部干预的时机要做出正确判断^[29]^。假进展和超进展均表现为经免疫治疗后病灶体积不减反增。假进展表现为先增后减的过程，复查发现肿瘤增大，随后再次复查发现肿瘤缩小，患者仍然可从免疫治疗中获益^[30]^。超进展则提示预后不佳，通常表现为在第一次评价时进展或至治疗失败时间 < 2个月、肿瘤体积增加 > 50%、肿瘤增长速度增加 > 2倍^[31, 32]^。而MR是部分病灶缓解，出现新病灶或有限的靶病灶增大，通常出现在对免疫治疗初始有应答的患者中。

### 3.2 出现MR情况，治疗方案是否需要改变

免疫治疗后出现MR，如果患者只是出现影像学上可被检测到的局部病灶的增大，全身肿瘤负荷可控，并没有明显的证据提示体能状态评分（performance status, PS）恶化，可以认为免疫治疗有效，全身治疗方案可不做改变，优先进行放疗局部干预。在对MR采取局部干预或考虑局部干预措施时，需要考虑病灶的部位、大小以及患者对治疗策略的耐受。同一器官的MR可能是肿瘤细胞本身的免疫原性存在异质性导致，不同器官之间的MR可能与所处的器官特异性的肿瘤微环境相关^[33]^。肾上腺、肺部单发结节或者脑部病灶局部干预比较理想，肝脏部分出现的MR局部干预效果较差，这可能与肝脏作为免疫耐受器官的免疫微环境的特殊性有关^[34]^，同时影像评估易受肝脏活动度影响。

如果患者肿瘤负荷快速增加或全身症状较严重，以及时更换全身治疗方案为首要原则。不论是局部治疗还是改变全身治疗方案，都需考虑患者的症状，症状往往是判断疾病是否进展，患者是否获益的重要指征。如果患者是真进展而非假进展，譬如：患者症状加重、肿瘤标志物水平升高等，建议及时进行相应的干预。

### 3.3 局部治疗干预方式选择

免疫治疗后出现MR的应对措施主要借鉴靶向治疗中的经验。局部治疗的方式包括放疗、手术治疗等，但在选择治疗方案时必须了解MR出现的机制。多个不同进展情况的病灶优先考虑放疗；孤立进展病灶，且位于可手术区域的病灶优先考虑手术；寡转移（远端转移在单一器官出现1个-5个病灶）应针对原发灶进行照射（放疗）^[30]^；弥散性的转移灶处理原则，学界尚缺少共识，临床研究^[35]^提示对原发灶进行放疗可一定程度改善临床结局，可考虑对原发肺部病灶进行高剂量照射。

此外，还需将病灶所在的器官纳入考量，比如针对肝脏的病灶，放疗可能不是最佳选择，可以考虑介入治疗。根据已知的研究，ICIs单药治疗对原发性肝癌和转移性肝癌的效果不佳，需要联合其他药物进行治疗，可优先考虑抗血管生成药物的联合治疗。

### 3.4 小结

对于接受ICIs治疗出现MR/混合进展，不改变全身治疗的方案，而采取放疗、手术治疗等局部治疗的方式，以患者可以耐受，体力评分良好，病灶进展数量和范围可控为前提。首先对MR进行鉴别诊断，对病灶出现的时间和性质进行定义，区分寡进展/寡恶化（1个-5个进展/恶化病灶）或广泛进展（> 5个进展病灶）。只有在患者疾病控制较好，出现寡进展/寡恶化时考虑局部治疗的干预。对于弥散转移/多发转移的病例，仍然需要最大化免疫全身治疗疗效后考虑局部处理。根据病灶数量和所在器官制定方案，比如对肝脏转移灶的干预，应该优先考虑联合抗血管生成治疗。

## 4 放疗联合免疫治疗脑转移的前沿问题探讨

颅内转移是晚期恶性肿瘤严重的不良预后因素，同时严重影响患者生活质量。越来越多的证据提示针对出现症状的脑转移患者主要以局部放疗为主，对无症状的脑转移患者则以全身治疗为主。靶向治疗对驱动基因阳性的患者具有良好的颅内控制率，而驱动基因阴性的患者仍依赖系统性化疗，由于化疗药物很难通过血脑屏障，这部分患者人群的脑转移治疗疗效不尽如人意，颅内成为转移瘤的避难所。随着免疫治疗时代来临，放疗与免疫治疗对颅内肿瘤的协同效应也成为临床和科研的热门话题。ICIs对放疗增敏作用、最佳治疗模式、联合治疗的安全性、以及对颅外病灶的远隔效应都值得探讨。

### 4.1 放疗联合免疫治疗激活颅内免疫系统

放射外科治疗（stereotactic radiosurgery, SRS）或全脑放疗（whole brain radiation therapy, WBRT）联合ICIs的协同增效具有理论基础^[36]^。基础和转化研究的相关结果显示免疫联合放疗具有协同作用。长期以来，学界认为大脑是一个免疫豁免器官，中枢神经系统（central nervous system, CNS）中没有淋巴管。2015年6月，*Nature*杂志发表了一项突破性科学进展研究^[37]^，研究团队寻找T细胞进出脑膜的通道时，发现了沿硬膜窦排列的功能性淋巴管。这些结构表达了淋巴管内皮细胞的所有分子特征，能够携带来自脑脊液的液体和免疫细胞，并与颈深淋巴结相连。CNS淋巴系统的发现为重新评估神经免疫学的基本假设提供了重要的基础，为肿瘤脑转移的机制和治疗策略研究提供了重要的突破。可以推测ICIs可以激发免疫活性T细胞（能够识别肿瘤抗原的T细胞）通过脑膜淋巴管系统和颈深淋巴结与颅内CNS免疫系统交互^[38]^。放疗有免疫刺激作用，而免疫治疗有“拖尾效应”，一旦起效可减少复发长期生存，两者联合可突破脑转移瘤避难所，因此有放疗指征的脑转移患者应积极考虑联合免疫治疗^[39]^。

### 4.2 放疗联合免疫治疗的最佳治疗模式

#### 4.2.1 联合治疗指征

患者有放疗指征即可进行联合治疗，2015年美国前总统卡特确诊晚期黑色素瘤时已90岁且合并4个颅内转移病灶，SRS联合免疫治疗后多年未复发^[40]^。

#### 4.2.2 最佳剂量

患者没有明显不良反应时，应按照原有剂量治疗。不建议在没有足够证据的情况下，降低放射外科治疗的剂量，因为SRS疗效比较确切，而且无法预测剂量降低后放疗疗效。全脑放疗常规剂量30 Gy/10 f，可能产生远期不良反应，联合免疫治疗可以考虑降低剂量。专家讨论组提出应针对低剂量放疗与ICIs的联合治疗展开进一步的探索，使用小于姑息照射剂量1/2的剂量，就能达到免疫增效的效果，能够促使甚至撬动免疫治疗的最大效能。如果保证疗效的前提下能够降低全脑照射的剂量，联合治疗将会大幅度减少不良反应，改善长期存活患者的认知功能和生活质量。

#### 4.2.3 治疗方式

治疗方式的选择需考虑以下几个方面：①如果患者有放射外科指征，尤其是4个以下的颅内病灶，首选大剂量的分割放疗快速杀伤肿瘤细胞；②如果患者没有放射外科指征，但是颅内肿瘤较大（> 5 cm）并伴随症状，应该积极考虑全脑照射；③对于没有伴随症状、病灶较小或弥散性病灶、驱动基因阳性的人群，目前倾向于使用靶向治疗，推迟放疗的干预，对于无驱动基因可以接受免疫系统治疗的人群，颅脑放疗干预仍是目前的标准，可进一步通过随机或前瞻性的研究探讨时间和剂量的把握。

#### 4.2.4 联合治疗时序

放疗与免疫治疗联合应用时，时序安排可能是获得最佳效果的另一关键因素。考虑到放射外科治疗周期极短，1个-4个病灶大约只需要1 d-2 d，放疗可以在免疫治疗的多个阶段进行，基本不存在延误治疗的问题。临床实践中倾向于放疗后使用免疫治疗，但需要注意ICIs联合应用后可能加重局部反应。放疗后使用免疫治疗延续局部炎症反应有助于肿瘤消退，然而炎症反应加重时需注意对正常脑组织的损伤。

### 4.3 免疫治疗联合颅脑放疗未明显增加中枢神经系统不良反应

目前没有证据证实免疫治疗联合放疗会增加不良反应^[41, 42]^，也没有证据表明联合治疗对脑转移患者相比无脑转移患者的不良反应率更高。MD Anderson肿瘤中心针对部分脑转移的患者进行免疫联合放疗的治疗，张玉蛟教授从现有尚未发表的数据来看，免疫联合放疗相对于单纯放疗，无论是采用全脑放疗还是立体定向放疗都未明显增加脑水肿和放射性脑坏死的发生率，毒性反应可耐受，没有出现预期以外的不良反应，亟待对现有数据深入分析以及进行前瞻性研究，提供更多可信数据，是未来研究的一个切入点。2019年*Lung Cancer*刊登的一项研究^[43]^对Ⅲb期/Ⅳ期非鳞NSCLC患者至少一种全身性治疗后进展后使用免疫治疗进行回顾性分析，1, 588例患者中409例伴有脑转移，其中242例脑转移患者接受了颅内照射治疗。脑转移组患者与总体患者相比，治疗相关不良反应无明显增多，所有级别不良反应发生率分别为35%和33%，3级-4级不良反应发生率分别为7%和6%，未观察到脑水肿和放射性脑坏死，显示联合治疗未明显增加脑水肿和放射性脑坏死的发生率。免疫联合放疗的安全性值得肯定^[44]^，尤其SBRT对颅内肿瘤的局部控制率高且对正常组织损伤小，对正常淋巴细胞分布和淋巴管分布影响小。

### 4.4 免疫治疗联合颅脑放疗促进系统抗肿瘤治疗的疗效

专家讨论组提出，颅内照射引起肿瘤细胞崩解释放新抗原，通过颅脑的淋巴管网系统向外输送到全身，从而引起远隔效应的假说，仍需进一步研究明确。MD Anderson肿瘤中心通过临床研究与临床实践积累了数百例相关病例，发现这种远隔效应对颅外病灶有一定控制率，但缓解率次于直接照射颅外病灶，这一结果将进行系统分析发表，提供更多研究数据，填补相关研究空白。目前远隔效应的研究多基于对肺部或肝脏病灶的直接照射进行分析。对颅内照射与系统免疫治疗的联合进行优化，促进远隔效应以提高整体疗效将是具有前瞻性的临床问题。

### 4.5 小结

ICIs联合放疗治疗脑转移可提高疗效并且未明显增加中枢系统不良反应^[36, 45]^，患者有放疗指征即可进行联合治疗，首选大剂量分割放疗，不建议在没有足够证据的情况下降低放射外科治疗的剂量。使用全脑放疗治疗颅脑多发转移或瘤体直径较大，有明显恶心、呕吐或下肢瘫痪时需要考量放射剂量是否应减量。对于有多发转移并且没有症状的颅内转移患者，未来需要研究探讨最佳的联合策略，包括联合的时序和放疗/ICIs治疗的把控。

## 5 总结与展望

放疗联合免疫治疗疗效明确，安全可控。尽管仍有不少争议性问题，随着临床应用的增多争议或将逐渐明朗，同时有更多新的问题显现。临床应用的演进和技术的进步使免疫的激活和启动最大化，并减少治疗相关的不良反应。随着全身治疗疗效的提升，患者生存期得到延长，对局部治疗的需求也将增高，两者相辅相成。全身治疗疗效的提高增加了局部治疗的重要性，更需要研究如何将局部治疗与全身治疗联合起来，达到最优的效果。张玉蛟教授提出免疫治疗联合立体定向消融放疗（immunotherapy-stereotactic ablative radiotherapy, I-SABR）概念，认为对早期患者应用I-SABR是今后的研究方向，虽然目前放疗联合免疫治疗主要应用于局部晚期NSCLC，但是考虑早期肺癌患者身体状况好，免疫状况好，肿瘤负荷相对小，有可能获得更好的临床收益。未来随着临床证据增多，放疗联合免疫治疗逐步有机会在更早阶段参与到肿瘤患者的治疗中。

## References

[1] Antonia SJ, Villegas A, Daniel D (2018). Overall survival with durvalumab after chemoradiotherapy in stage Ⅲ NSCLC. N Engl J Med.

[2] Antonia SJ, Villegas A, Daniel D (2017). Durvalumab after chemoradiotherapy in stage Ⅲ non-small-cell lung cancer. N Engl J Med.

[3] Gray JE, Villegas A, Daniel D (2020). Three-year overall survival with durvalumab after chemoradiotherapy in stage Ⅲ NSCLC-update from PACIFIC. J Thorac Oncol.

[4] Theelen W, Peulen HMU, Lalezari F (2019). Effect of pembrolizumab after stereotactic body radiotherapy vs. pembrolizumab alone on tumor response in patients with advanced non-small cell lung cancer: Results of the PEMBRO-RT phase 2 randomized clinical Trial. JAMA Oncol.

[5] Lazzari C, Karachaliou N, Bulotta A (2018). Combination of immunotherapy with chemotherapy and radiotherapy in lung cancer: is this the beginning of the end for cancer?. Ther Adv Med Oncol.

[6] Brooks ED, Chang JY (2019). Time to abandon single-site irradiation for inducing abscopal effects. Nat Rev Clin Oncol.

[7] Citrin DE (2017). Recent developments in radiotherapy. N Engl J Med.

[8] Gong X, Li X, Jiang T (2017). Combined radiotherapy and anti-PD-L1 antibody synergistically enhances antitumor effect in non-small cell lung cancer. J Thorac Oncol.

[9] Chicas-Sett R, Morales-Orue I, Castilla-Martinez J (2019). Stereotactic ablative radiotherapy combined with immune checkpoint inhibitors reboots the immune response assisted by immunotherapy in metastatic lung cancer: a systematic review. Int J Mol Sci.

[10] Postow MA, Callahan MK, Barker CA (2012). Immunologic correlates of the abscopal effect in a patient with melanoma. N Engl J Med.

[11] Wang H, Lin X, Luo Y (2019). alpha-PD-L1 mAb enhances the abscopal effect of hypo-fractionated radiation by attenuating PD-L1 expression and inducing CD8(+) T-cell infiltration. Immunotherapy.

[12] Perez-Gracia JL, Labiano S, Rodriguez-Ruiz ME (2014). Orchestrating immune check-point blockade for cancer immunotherapy in combinations. Curr Opin Immunol.

[13] Shaverdian N, Lisberg AE, Bornazyan K (2017). Previous radiotherapy and the clinical activity and toxicity of pembrolizumab in the treatment of non-small-cell lung cancer: a secondary analysis of the KEYNOTE-001 phase 1 trial. Lancet Oncol.

[14] Durm GA, Althouse SK, Sadiq AA (2018). Phase Ⅱ trial of concurrent chemoradiation with consolidation pembrolizumab in patients with unresectable stage Ⅲ non-small cell lung cancer: Hoosier Cancer Research Network LUN 14-179. J Clin Oncol.

[15] Lin SH, Lin Y, Yao L (2020). Phase Ⅱ trial of concurrent atezolizumab with chemoradiation for unresectable NSCLC. J Thorac Oncol.

[16] Postow MA, Sidlow R, Hellmann MD (2018). Immune-related adverse events associated with immune checkpoint blockade. N Engl J Med.

[17] Hwang WL, Pike LRG, Royce TJ (2018). Safety of combining radiotherapy with immune-checkpoint inhibition. Nat Rev Clin Oncol.

[18] Tian S, Switchenko J, Patel P (2019). MA01. 02 lung stereotactic body radiotherapy and concurrent immunotherapy: A multi-center safety and toxicity analysis. J Thorac Oncol.

[19] Antonia SJ, Balmanoukian A, Brahmer J (2019). Clinical activity, tolerability, and long-term follow-up of durvalumab in patients with advanced NSCLC. J Thorac Oncol.

[20] Chun SG, Hu C, Choy H (2017). Impact of intensity-modulated radiation therapy technique for locally advanced non-small-cell lung cancer: A secondary analysis of the NRG oncology RTOG 0617 randomized clinical trial. J Clin Oncol.

[21] Bradley JD, Paulus R, Komaki R (2015). Standard-dose versus high-dose conformal radiotherapy with concurrent and consolidation carboplatin plus paclitaxel with or without cetuximab for patients with stage Ⅲa or ⅢB non-small-cell lung cancer (RTOG 0617): a randomised, two-by-two factorial phase 3 study. Lancet Oncol.

[22] Faehling M, Schulz C, Laack H (2019). PACIFIC subgroup analysis: pneumonitis in stage Ⅲ, unresectable NSCLC patients treated with durvalumab *vs*. placebo after CRT. Pneumologie.

[23] Lin SH, Lin Y, Mok I (2019). Phase Ⅱ trial combining atezolizumab concurrently with chemoradiation therapy in locally advanced non-small cell lung cancer. J Clin Oncol.

[24] Dovedi SJ, Adlard AL, Lipowska-Bhalla G (2014). Acquired resistance to fractionated radiotherapy can be overcome by concurrent PD-L1 blockade. Cancer Res.

[25] Baldeo C, Kaleem T, Paz-Fumagalli R (2019). Mixed response to immunotherapy in lung cancer. J Clin Oncol.

[26] Baldeo C, Kaleem T, Paz-Fumagalli R (2019). CLO19-026: Determining the treatment decision of tumors with mixed response. J Natl Compr Canc Netw.

[27] Osorio JC, Arbour KC, Le DT (2019). Lesion-level response dynamics to programmed cell death protein (PD-1) blockade. J Clin Oncol.

[28] Seymour L, Bogaerts J, Perrone A (2017). iRECIST: guidelines for response criteria for use in trials testing immunotherapeutics. Lancet Oncol.

[29] Borcoman E, Kanjanapan Y, Champiat S (2019). Novel patterns of response under immunotherapy. Ann Oncol.

[30] Ferrara R, Caramella C, Besse B (2019). Pseudoprogression in non-small cell lung cancer upon immunotherapy: Few drops in the ocean?. J Thorac Oncol.

[31] Frelaut M, Le Tourneau C, Borcoman E (2019). Hyperprogression under immunotherapy. Int J Mol Sci.

[32] Ferrara R, Mezquita L, Texier M (2018). Hyperprogressive disease in patients with advanced non-small cell lung cancer treated with PD-1/PD-L1 inhibitors or with single-agent chemotherapy. JAMA Oncol.

[33] Garelli E, Rittmeyer A, Putora PM (2019). Abscopal effect in lung cancer: three case reports and a concise review. Immunotherapy.

[34] Tumeh PC, Hellmann MD, Hamid O (2017). Liver metastasis and treatment outcome with anti-PD-1 monoclonal antibody in patients with melanoma and NSCLC. Cancer Immunol Res.

[35] Shiarli A-M, McDonald F, Gomez D (2019). When should we irradiate the primary in metastatic lung cancer?. Clin Oncol (R Coll Radiol).

[36] Shepard MJ, Xu Z, Donahue J (2019). Stereotactic radiosurgery with and without checkpoint inhibition for patients with metastatic non-small cell lung cancer to the brain: a matched cohort study. J Neurosurg.

[37] Louveau A, Smirnov I, Keyes TJ (2015). Structural and functional features of central nervous system lymphatic vessels. Nature.

[38] Kamath SD, Kumthekar PU (2018). Immune checkpoint inhibitors for the treatment of central nervous system (CNS) metastatic disease. Front Oncol.

[39] Petrelli F, De Stefani A, Trevisan F (2019). Combination of radiotherapy and immunotherapy for brain metastases: A systematic review and *meta*-analysis. Crit Rev Oncol Hematol.

[40] Gampa G, Vaidhyanathan S, Wilken-Resman B (2016). Challenges in the delivery of therapies to melanoma brain metastases. Curr Pharmacol Rep.

[41] Goldberg SB, Gettinger SN, Mahajan A (2016). Pembrolizumab for patients with melanoma or non-small-cell lung cancer and untreated brain metastases: early analysis of a non-randomised, open-label, phase 2 trial. Lancet Oncol.

[42] Goldberg SB, Gettinger SN, Mahajan A (2018). Durability of brain metastasis response and overall survival in patients with non-small cell lung cancer (NSCLC) treated with pembrolizumab. J Clin Oncol.

[43] Crinò L, Bronte G, Bidoli P (2019). Nivolumab and brain metastases in patients with advanced non-squamous non-small cell lung cancer. Lung Cancer.

[44] Arscott WT, Zhu S, Plastaras JP (2019). Acute neurologic toxicity of palliative radiotherapy for brain metastases in patients receiving immune checkpoint blockade. Neurooncol Pract.

[45] Chen L, Douglass J, Kleinberg L (2018). Concurrent immune checkpoint inhibitors and stereotactic radiosurgery for brain metastases in non-small cell lung cancer, melanoma, and renal cell carcinoma. Int J Radiat Oncol Biol Phys.

